# Rad26, the Transcription-Coupled Repair Factor in Yeast, Is Required for Removal of Stalled RNA Polymerase-II following UV Irradiation

**DOI:** 10.1371/journal.pone.0072090

**Published:** 2013-08-21

**Authors:** Sounak Ghosh-Roy, Dhiman Das, Debarati Chowdhury, Michael J.Smerdon, Ronita Nag Chaudhuri

**Affiliations:** 1 Department of Biotechnology, St. Xavier’s College, Mother Teresa Sarani, Kolkata, India; 2 Biochemistry and Biophysics, School of Molecular Biosciences, Washington State University, Pullman, Washington, United States of America; Florida International University, United States of America

## Abstract

Transcription coupled nucleotide excision repair (TCR) is a major pathway responsible for removal of helix distorting DNA lesions from transcriptionally active regions of the genome. Rad26, a key factor of the TCR pathway, is known to play a role during early steps of TCR. Here, we show that Rad26-mediated TCR is not absolutely dependent on active transcription elongation in budding yeast. As per our results, *RAD26-*deleted cells show enhanced UV sensitivity compared to wild type cells under conditions where transcription elongation is inhibited. The increased UV sensitivity observed in *RAD26*-deleted cells, however, is not due to reduced expression of the major NER-responsive genes. Interestingly, transcription of the constitutively expressed *RPB2* gene is adversely affected in *RAD26-*deleted cells during UV-induced DNA damage repair. In consonance, chromatin immunoprecipitation analysis showed that unlike in wild type, in *RAD26*-deleted cells no significant reduction in RNA polymerase II occupancy occurs during nucleotide excision repair in the transcriptionally active loci like, *RPB2*, *PYK1* and *RPL2B*. These results collectively indicate that removal of RNAPII during DNA damage repair following UV irradiation is dependent on Rad26.

## Introduction

Stability of the eukaryotic and prokaryotic genome is continually challenged by various exogenous and endogenous sources that can damage DNA. Such damages might lead to mutations, impairment of DNA metabolic processes and finally to cell death, if not corrected. The UV-induced lesions like cyclobutane pyrimidine dimers (CPD) or 6-4 photoproducts, and other helix distorting DNA adducts caused by agents like cisplatin and 4-nitroquinoline-1-oxide, are of considerable concern as they can block transcription. It is known that cells have evolved a repair mechanism called the nucleotide excision repair (NER) pathway to combat such helix distorting lesions and minimize their inhibitory effect on transcription. NER is known to have two sub-pathways: (a) global genome NER or GGR, which repairs lesions located in the transcriptionally silent or inactive regions of the genome; and (b) transcription-coupled NER or TCR, which repairs lesions located in the transcribed strands of active genes [1], [2], [3]. The GGR and TCR pathways are fundamentally same except at the damage recognition step [4]. While damage recognition is known to be the rate-limiting step of NER, TCR is initiated when the RNA polymerase II complex stalls at a DNA lesion and the subsequent repair machinery acts rapidly to remove lesions from the transcribed strand [5], [6], [7], [8]. It has been shown that the RNA polymerase II complex interacts with DNA containing a CPD and, in fact, makes the lesion less accessible [9], [10]. Furthermore, on encountering CPD lesions the conformation of RNA Pol II does not change [10]. Therefore, following such encounter of DNA damage with RNA Pol II, TCR requires an additional mechanism to render the DNA lesion accessible to the NER factors.

Rad26, the yeast homolog of human Cockayne Syndrome B (CSB) protein, has been implicated to be a primary factor involved in the early steps of TCR. Although evidence for a Rad26-independent TCR pathway also exists [3], [11], [12], [13], it is known that deletion of Rad26 renders yeast cells defective in TCR [11]. Studies have also indicated a role of Rad26 in transcription elongation [14], [15], [16]. CSB and Rad26 are both members of the Swi2/Snf2 family with DNA-dependent ATPase domains, which can change DNA accessibility to proteins by altering chromatin structure [17], [18], [19]. It has been suggested that CSB monitors the progression of RNAPII and stable recruitment occurs when a DNA lesion is encountered [19], thereafter the chromatin structure is altered through interaction with histone tails [20]. In bacteria, Mfd, the designated transcription-repair coupling factor, is known to remove RNA polymerase complex stalled at DNA lesions and mediate recruitment of subsequent NER factors [21], [22], [23]. In eukaryotes, however, the exact steps of CSB/Rad26 mediated TCR remain to be elucidated. CSB is unable to displace RNAPII from the lesion site [17], and although the Swi/Snf like enzymes in eukaryotes are known to possess DNA translocase activity, no direct evidence for the translocase activity or recruitment of NER factors to the lesion site by Rad26 has been demonstrated thus far. It has been suggested that Rad26 works to alter the RNAPII-DNA contact site and somehow removes RNAPII from the lesion site so that subsequent repair and resumption of transcription can occur [7]. Furthermore, Woudstra and colleagues [24] have identified a novel protein (Def1) associated with Rad26 that plays a role in DNA damage response as well as transcription elongation. The authors have shown that following UV-irradiation, Def1 mediates RNAPII degradation via the ubiquitination-mediated protein degradation pathway. This is in consonance with the work done by Sharp and colleagues [25] who showed that arrested RNAPII is a target of ubiquitination *in vitro*. Conversely, Rad26 seems to work in a manner opposite to Def1, as deletion of Rad26 leads to rapid degradation of RNAPII following UV irradiation in yeast cells [24].

Earlier, we have shown that *RAD26*- deleted yeast strains show decreased TCR in the transcriptionally active locus *RPB2*, but this deletion did not significantly affect repair in the transcriptionally silent locus *HML* [26]. The present investigation was undertaken to further elucidate the role of Rad26 during TCR. We observed that Rad26-mediated TCR is not solely dependent on active transcription elongation, as even under conditions where transcription elongation is impaired, *RAD26*- deleted cells show enhanced UV sensitivity compared to wild type cells. As deletion of Rad26 did not affect expression of common NER genes, the increased UV sensitivity of *RAD26*-deleted cells is possibly not due to reduced availability of NER factors. Deletion of *RAD26* however affects transcription of the constitutively expressed *RPB2* gene following UV-induced DNA damage repair. Chromatin immunoprecipitation (ChIP) analyses showed loss of RNAPII in the different ORF regions of constitutively expressed loci such as, *RPB2, PYK1* and *RPL2B*, post UV irradiation. However, similar reduction in RNAPII occupancy was not observed in the *RPB2, PYK1* and *RPL2B* loci of UV-irradiated *RAD26*-deleted cells. Our work provides evidences for a role of Rad26 in removal of RNAPII during TCR, in the constitutively active regions of budding yeast cells.

## Materials and Methods

### UV Sensitivity Assay

For UV sensitivity assay, cells were diluted to different concentrations, spread on YPD plates and irradiated with the indicated UV doses. Colonies were counted after 48 h of incubation at 30°C in the dark, as described in [26], [27].

### MPA and 6-azauracil Sensitivity Assay

For MPA and 6-azauracil sensitivity assays, yeast cells of ∼0.6 OD_600_ were spread on SC plates containing 15, 30 and 45 µg/ml of MPA or 25, 50, 100 and 150 µg/ml of 6-azauracil respectively. Colonies were counted after 72 h of incubation at 30°C. For UV sensitivity assays in presence of transcription elongation inhibitors yeast cells of ∼0.6 OD_600_ were spread on SC plates containing 30 µg/ml of MPA or 100 µg/ml of 6-azauracil respectively and irradiated with UV doses, as indicated. Colonies were counted after 72 h of incubation at 30°C at dark.

### RT–PCR Analysis

For analysis of NER specific genes, cells were grown to log phase in YPD and treated without or with 100 J/m^2^ of UV radiation and allowed to repair for 1 h. Total RNA was isolated from each yeast culture and ∼5 µg of RNA from each sample was reverse-transcribed using Revert Aid Reverse Transcriptase (Fermentas, USA), as per manufacturer’s instructions. PCR amplification was done for 25 cycles using gene specific primers. For transcriptional analysis of *RPB2* gene, the cells were UV irradiated at 100 J/m^2^ and allowed to repair for different time periods, as indicated.

### Chromatin Immunoprecipitation

Chromatin immunoprecipitation (ChIP) was performed as described [27]. Mid-log phase yeast cells were treated with or without 100 J/m^2^ UV and allowed to repair for indicated time. Cells were then crosslinked with 1% formaldehyde and after suspension in lysis buffer (50 mM HEPES, pH 7.5, 140 mM NaCl, 1 mM EDTA, 1% Triton X-100, 0.1% sodium deoxycholate, 1 mM PMSF, 1 mg/ml leupeptin, 1 mg/ml pepstatin A) were disrupted using glass beads (425–600 µm, Sigma), followed by sonication. Protein levels in the extract were estimated using the Bradford assay. Equal amounts of protein from each sample were used for immunoprecipitation with anti-RNA Polymerase II monoclonal antibody 8WG16 (Covance: MMS-126R). The reaction mixture was incubated overnight at 4°C, and the immunocomplex precipitated using Protein A sepharose beads (50% slurry). The beads were consecutively washed with lysis buffer, wash buffer 1 (Lysis buffer containing 500 mM NaCl), wash buffer 2 (10 mM Tris–HCl, pH 8, 250 mM LiCl, 0.5% NP-40, 0.5% sodium deoxycholate, 1 mM EDTA) and TE buffer and then treated with RNase A in TE at 37°C for 30 min. Chromatin was then eluted from the beads using elution buffer (1% SDS, 0.1 M NaHCO_3_) and the crosslinks reversed by incubation at 65°C overnight. Fragments representing specific ORF regions of *RPB2* locus were amplified from the immunoprecipitated DNA using sequence-specific primers. The forward and reverse primer sets used for ORF1, ORF2, and ORF3 of *RPB2* gene were 5′-ATGTCAGACCTTGCAAACTCAG-3′ and 5′- TTCGGTAGTATGTTGAGCCA-3′; 5′- TCAAGTCAAGCTTTATGGTCGT-3′ and 5′- AAGCATTTCCAGCATTTGCC-3′; 5′- TGGTCACACAGGTAAAAAACT-3′ and 5′- CCGAATCTTAAACCACCGTCTC-3′, respectively; for ORF1, ORF2, and ORF3 of *PYK1* gene were: 5′-CTCATTAAACGTTGTTGCTGG-3′ and 5′-ACCTGGGTACAATTCTTCGGA-3′; 5′-ATGGTTGCCAGAGGTGACTT-3′ and 5′-TACCGACATCGGAAACTTCA-3′; 5′-ATGCCCAAGAGCTGCTAGATT-3′ and 5′-ACTTGCAAAGTGTTGGAGTGA-3′ respectively; for ORF1, ORF2, and ORF3 of *RPL2B* gene were: 5′-TTACAAGGCAACATAGCAGCG-3′ and 5′-CCCTGTTGTCATGATACAAAA-3′; 5′-AAAGAAAGGGTGCTGGTTCT-3′ and 5′-AAACAACCTTGGCCAATGGA-3′; 5′-CAAACCATTGTTGAAGGCTG-3′ and 5′-TTGGGTCTTTTGAGAACCACG-3′ respectively. PCR products were resolved on 1.5% agarose gels. Experiments were repeated four times and the data is representative of the average of the different experimental sets.

## Results

In trying to understand the role of Rad26 during TCR, we deleted *RAD26* from both wild type as well as the Sin mutant H4 R45H cells. Sin mutants are Swi/Snf Independent mutants and repair studies have shown that the Sin mutant H4 R45H is more resistant to UV irradiations and have faster nucleotide excision repair rate compared to wild type cells [26]. Transcriptome analysis revealed that under normal conditions 475 genes are up-regulated in H4 R45H cells compared to wild type. H4 R45H cells show high rates of transcription coupled NER in the constitutively active *RPB2* locus. *RAD26* deletion have distinctly adverse effect on the NER rate of both wild type and H4 R45H cells, the effect being more profound on the latter. Here we have tried to further our understanding on the role of Rad26 during UV-induced DNA damage response and transcription coupled NER.

### UV Sensitivity of Rad26 Deleted Yeast Cells

**Figure 1:**
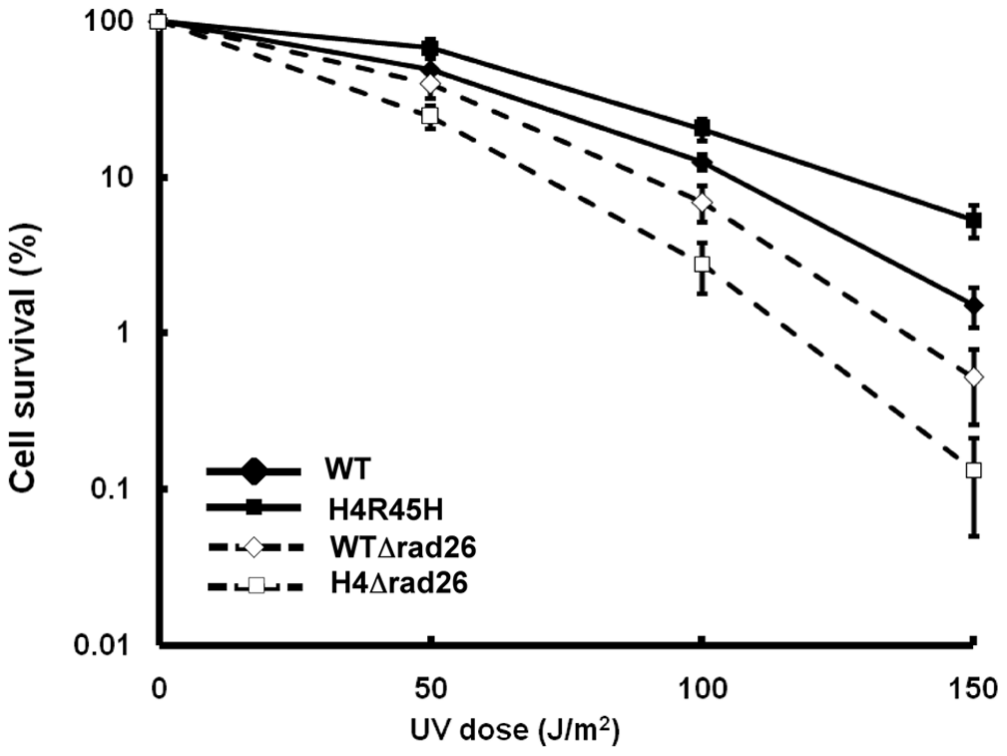
UV sensitivity of cells. UV sensitivity of WT, H4 R45H, WTΔRad26 and H4 R45HΔrad26 cells. Colony forming ability following UV irradiation was monitored in exponentially growing cultures. Cells were appropriately diluted, spread on YPD plates, subjected to the UV doses shown and their survival monitored. For each strain, data represent the mean ±1 SD for four independent experiments.

When subjected to UV irradiation, it was found that both *RAD26* -deleted wild type cells and *RAD26* -deleted H4 R45H cells show increased UV sensitivity compared to their Rad26-containing counterparts, respectively (Figure 1- compare solid to open diamond and solid squares to open squares). Of the strains tested, most affected were the *RAD26*-deleted H4 R45H cells (Figure 1). This result indicates that due to *RAD26* deletion while wild type cells are affected, the H4 R45H cells suffer more serious effects.

### Effect of Transcription Elongation Inhibitors on RAD26 -Deleted Cells

**Figure 2:**
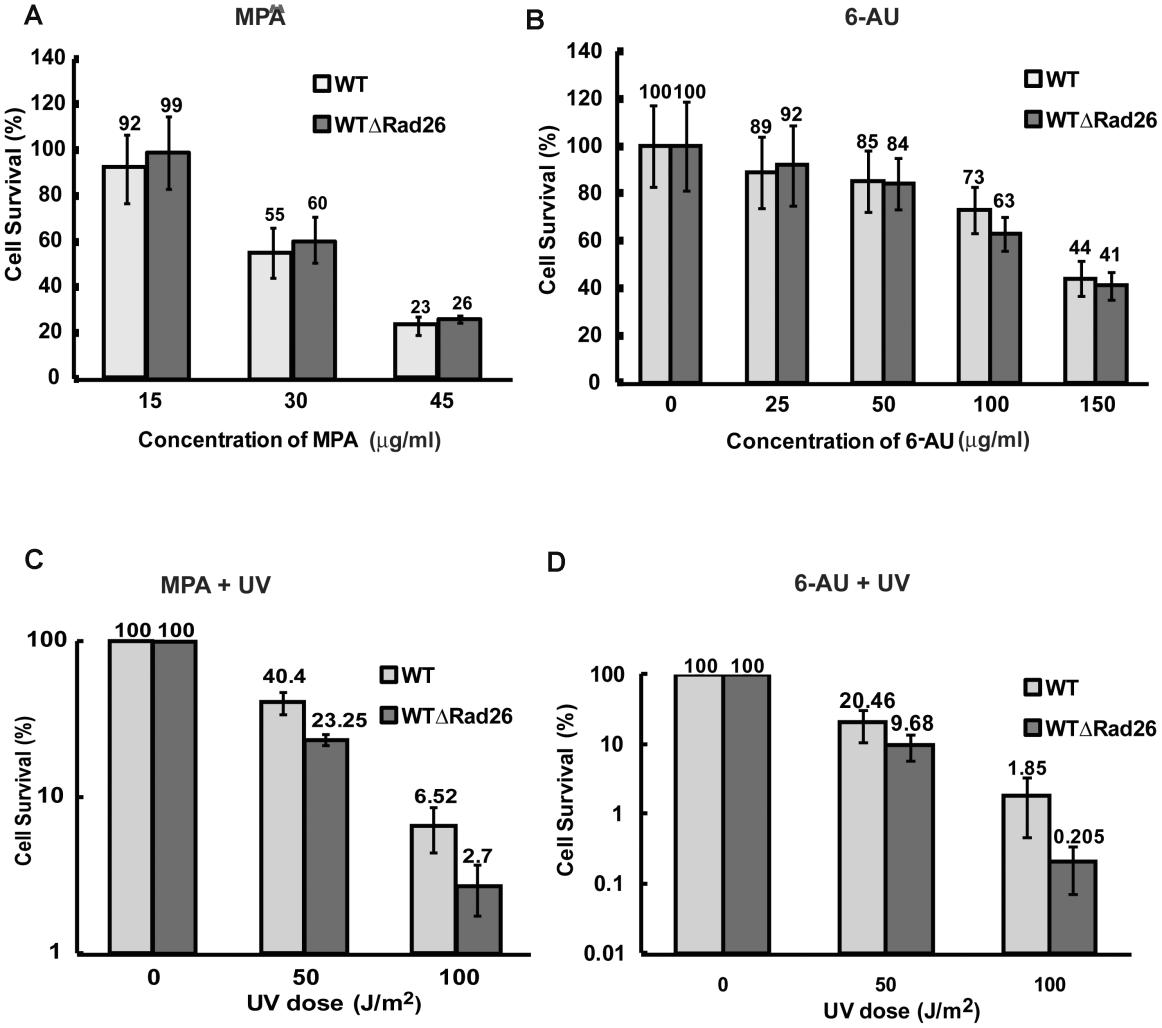
Sensitivity of cells to transcription elongation inhibitors with or without UV treatment. Cells of exponentially growing cultures were appropriately diluted and spread on SC plates supplemented with MPA (A) or 6-AU (B) of indicated concentrations without UV irradiation. Similarly grown cells were spread on SC plates supplemented with MPA (C) or 6-AU (D) and subjected to UV doses as indicated. Growth was monitored after 72 h. For each strain, data represent the mean ±1 SD for four independent experiments.

Several studies have indicated a role for Rad26 in transcription elongation [14], [16]. Therefore, we next tested whether Rad26-mediated TCR was absolutely dependent on active transcription elongation. For this we treated both wild type and *RAD26*-deleted cells with mycophenolic acid (MPA) and 6-azauracil (6-AU), both transcription elongation inhibitors. MPA, an inhibitor of IMP dehydrogenase is known to diminish the intracellular GTP pool, while 6-AU, an inhibitor of IMP dehydrogenase and orotidylate decarboxylase can diminish both GTP and UTP pools [28]. As shown in Figure 2A and 2B, when treated with MPA or 6-AU, wild type and *RAD26*-deleted cells showed comparable sensitivity. This indicate that lack of Rad26 do not confer additional sensitivity to transcription elongation inhibitors like MPA and 6-AU. However, when UV irradiated in presence of MPA or 6-AU, *RAD26*-deleted cells show considerable decrease in survivability compared to wild type (Figure 2C & 2D). It therefore implies that *RAD26* deletion adversely affects UV survivability of cells, irrespective of their transcription elongation status.

### RAD26 Deletion does not Reduce Expression of NER Factors but Affects RPB2 Transcription during NER

**Figure 3:**
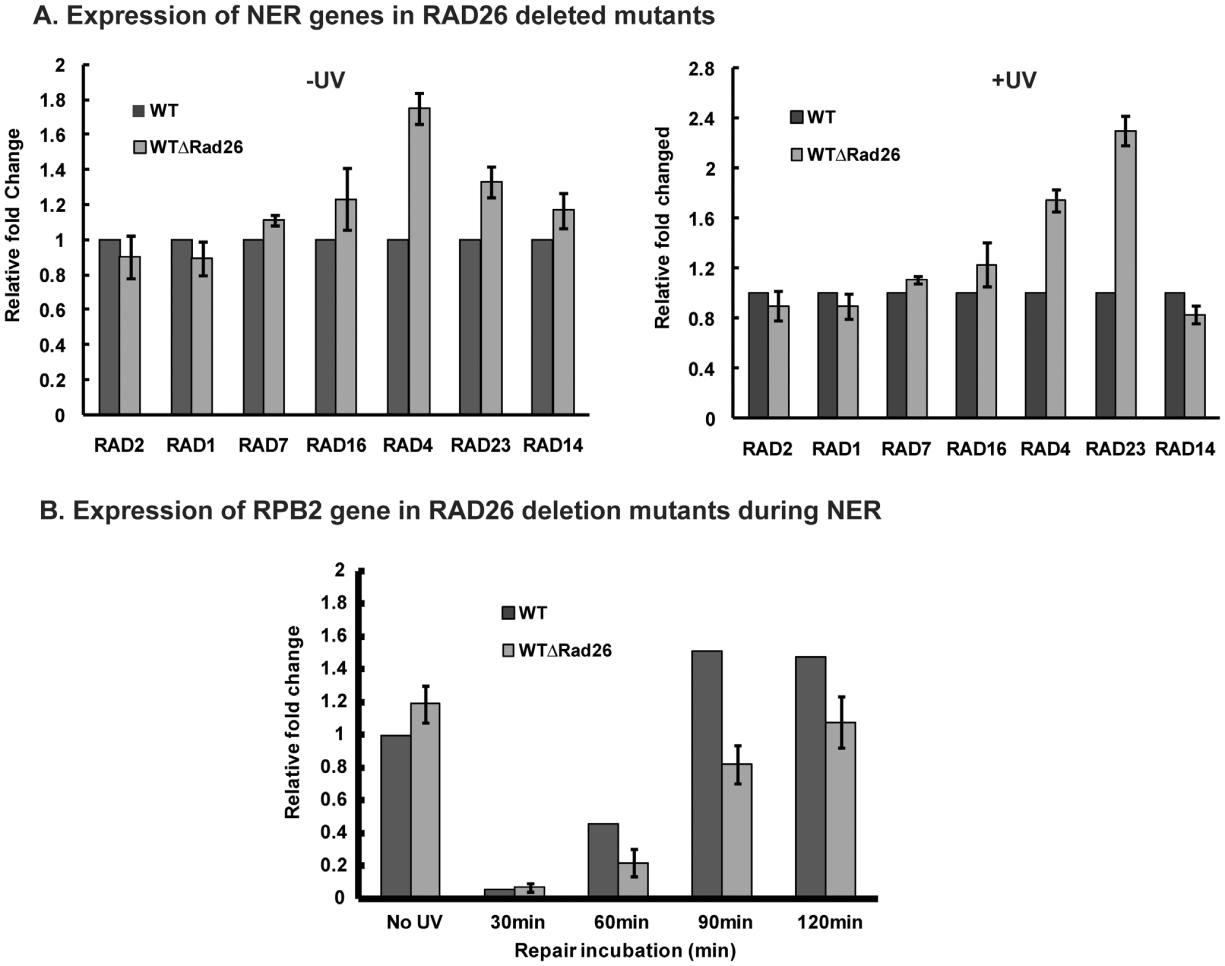
Expression analyses of NER genes and *RPB2* gene. RT–PCR analysis was performed on total RNA isolated from WT and WTΔRad26 cells (**A**) following treatment without or with 100 J/m^2^ UV radiation, using gene-specific primers, as described in Materials and Methods section; (**B**) of *RPB2* gene after 100 J/m^2^ UV irradiation followed by repair incubation for different time periods. For each strain, data represent the mean ±1 SD for three independent experiments.

To test whether increased UV sensitivity of *RAD26*-deleted cells was due to reduced expression of NER genes, transcriptional analyses of some NER-responsive genes were done in presence and absence of UV. As shown in Figure 3A, in absence or presence of UV irradiation, expression of the NER genes tested, namely, *RAD2*, *RAD1*, *RAD7*, *RAD16, RAD4, RAD23* and *RAD14* was not affected in *RAD26*-deleted cells. This indicates that impaired transcription of NER genes is possibly not the cause of increased UV sensitivity exhibited by *RAD26*-deleted cells.

We next tested the transcription rate of *RPB2* gene during NER and found that following UV irradiation and subsequent repair incubation, expression of the constitutively transcribed *RPB2* gene was reduced in *RAD26*-deleted cells compared to wild type (Fig. 3B). As shown in Figure 3B, significant difference in the expression level of *RPB2* gene was observed between wild type and *RAD26*-deleted cells after 60 min and 90 min of repair incubation. These results indicate that following UV irradiation, *RAD26* deletion significantly affects *RPB2* transcription.

### Fate of RNA Polymerase II during DNA Repair of the Constitutively Transcribing Loci

**Figure 4:**
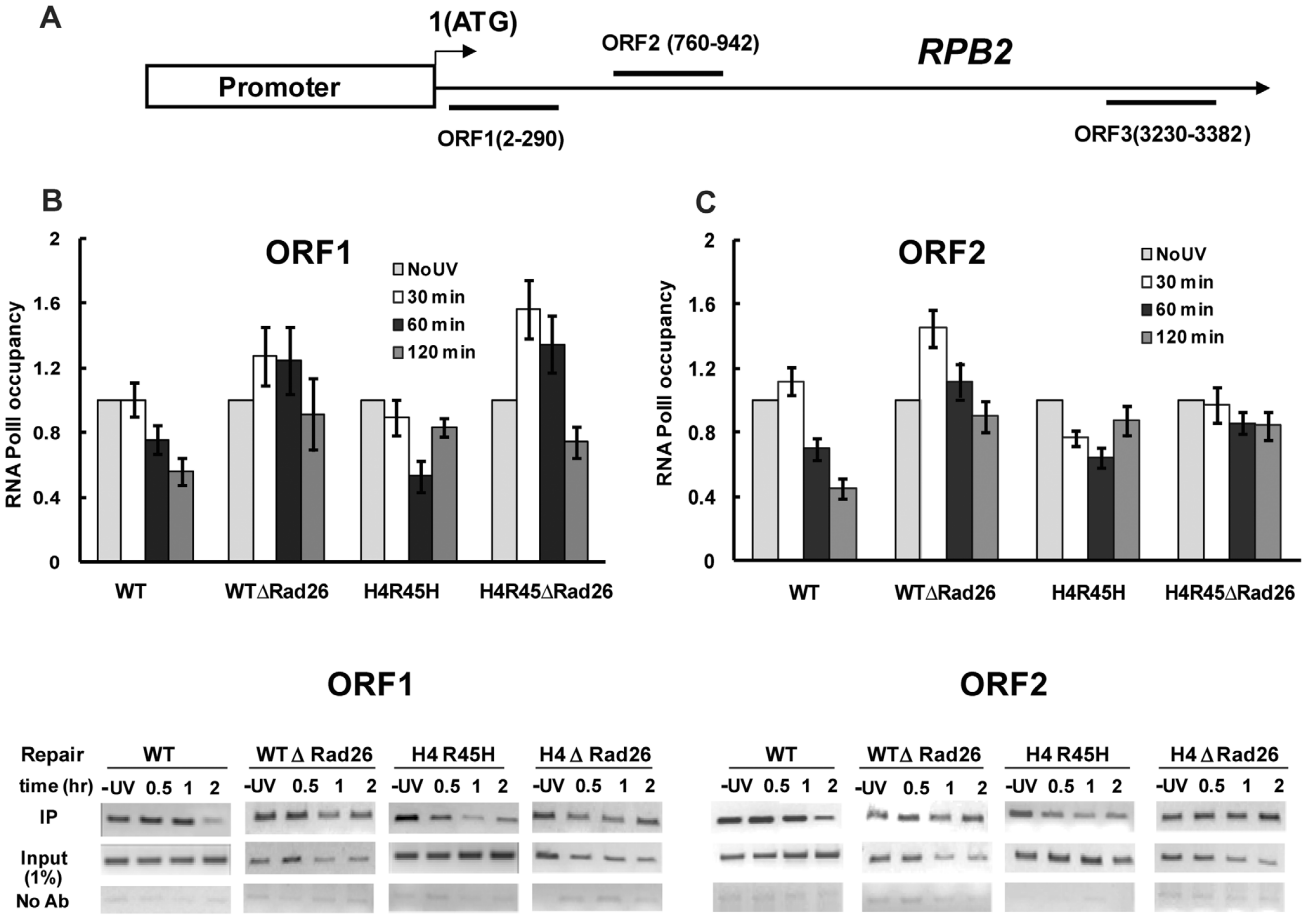
RNA polymerase II status during NER in different regions of the *RPB2* locus. ChIP analysis of RNA polymerase II status in different ORF regions of the *RPB2* locus as shown in (A), during NER. Cells were irradiated with 100 J/m^2^ UV and incubated for different repair times as indicated. Chromatin was immunoprecipitated with 8WG16 antibody specific to RNA polymerase II, followed by quantitative PCR amplification using primers specific to ORF1 (B), ORF2 (C) and ORF3 (data not shown) of the *RPB2* locus in WT, H4 R45H, WTΔRad26 and H4R45HΔRad26 cells. The values given for ORF1 and ORF2 are calculated by normalizing the ChIP -PCR signal with the input PCR signal. The value for UV untreated cells was set as 1.0. For each strain, data represent the mean ±1 SD for four independent experiments. Corresponding ChIP-PCR, input-PCR and no antibody control gel pictures are given below each strain.

It is known that TCR is initiated by stalled RNA polymerase II and that removal of this complex from the lesion site is a rate-limiting step for this process [4], [8]. Therefore, we next examined whether *RAD26* deletion affects removal of stalled RNAPII during NER, in both wild type and the H4R45H cells. For these experiments, we performed ChIP analyses with antibody (8WG16) against Rpb1, the largest subunit of RNAPII. We examined the level of RNAPII in different regions of the *RPB2* locus, with or without UV irradiation followed by repair incubation for different time periods. The presence of RNAPII was checked in three different ORF regions of the *RPB2* locus: ORF1 and ORF2 that are located near the 5′ end of the gene, and ORF3 located near the 3′ end of the gene (Fig. 4A). As seen in Figure 4B, ChIP-PCR in ORF1 indicates that RNAP II signal intensity decreases in both wild type and H4R45H cells during repair incubation. In wild type cells significant reduction of RNAPII signal intensity occurs during 60 min to 120 min of repair incubation. Interestingly, in the transcriptionally active genome of H4 R45H cells the RNAPII signal intensity starts to decrease early, i.e., after 30 min of repair and is significantly reduced within 60 min of repair. On the contrary, no significant reduction in RNAP II signal intensity was observed in the *RAD26*-deleted cells during repair incubation (Fig. 4B). Similar results were obtained for ORF2, where loss of RNAPII signal occurs in wild type and H4R45H cells within 60 min of repair (Fig. 4C). However, no reduction in RNAP II occupancy was observed for *RAD26*-deleted cells during NER. ChIP PCR done for ORF3 of *RPB2* locus, which is located towards the 3′ end of the gene, showed no significant RNAPII signal with the specific antibody used (8WG16) (Fig. S1). Lack of significant RNAPII signal in ORF3 is probably due to inability of the 8WG16 antibody to recognize RNA Pol II at the 3′ end of the genes due to certain RNA polymerase II carboxy-terminal domain modifications in late elongation phases, which often influence 8WG16 antibody recognition [29], especially when distance between the 5′ end and 3′ end of the gene is long, as in 3.7 kb *RPB2* locus.

To test whether the above phenomenon could be observed in other constitutively transcribed regions of our yeast strain as well, we performed similar ChIP-based experiment in the *PYK1* and *RPL2B* loci. The different ORF regions tested for *PYK1* and *RPL2B* are depicted in Figure S2A & S3A respectively. Results similar to *RPB2* were obtained for *PYK1* and *RPL2B* loci, as well. As shown in Figure S2B & S3B, in all the three ORFs tested in *PYK1* and *RPL2B*, we saw loss of RNAPII signals during NER, in both wild type and H4R45H cells. However, in *RAD26*-deleted cells no such reduction in RNAPII occupancy was observed in any of the ORFs of either *PYK1* or *RPL2B* loci. ORF3 regions of both *PYK1* (1.5 kb) and *RPL2B* (1.1kb) could be detected with the 8WG16 antibody probably due to shorter size of the loci.

## Discussion

We have earlier shown that TCR forms a major part of NER in Sin (Swi/Snf-independent) mutant H4 R45H cells, which have a distinctly faster NER rate compared to wild type. Deletion of the TCR factor Rad26 considerably decreased NER rate in the transcriptionally active loci of wild type and H4R45H cells, the effect being more drastic on the latter [26]. In the present work we have tried to gain further insight into the role of Rad26 during TCR, using both wild type and H4R45H Sin mutant cells.

### A More Actively Transcribing Yeast Strain Shows Enhanced UV Sensitivity in Absence of Rad26

When UV sensitivity of the *RAD26*-deleted cells was studied, it was observed that Rad26 deletion leads to higher UV sensitivity in both wild type and H4R45H cells (Fig. 1). Increased UV sensitivity exhibited by the *RAD26-*deleted cells compared to wild type cells, although small is consistent and reproducible. In light of the fact that Rad26-mediated TCR is restricted to the actively transcribing regions of the genome and that Rad26-independent TCR pathway also exists [3], [11], [12], [13], the small UV-sensitivity difference observed at the whole genome level is significant. Interestingly, the effect of *RAD26* deletion is more profound on the UV sensitivity of H4R45H mutant strain (Fig. 1) Our earlier work has shown that H4 R45H mutation leads to a more accessible chromatin structure and consequently more actively transcribing genome, where 475 genes are upregulated compared to wild type cells, under normal condition [26]. Thus, it may be considered that at any given point of time, the number of actively transcribing genes in the H4 R45H genome are higher compared to wild type [26]. Consequently, in such an actively transcribing genome of H4 R45H, following UV-irradiation, the need for TCR- that removes UV-induced DNA lesions from the actively transcribing strands, will presumably be greater, compared to wild type. Therefore, it is implicative that in absence of the TCR factor Rad26, H4 R45H cells are more seriously affected compared to wild type.

### Rad26 Mediated TCR is Primarily Dependent on Transcription Impaired due to UV-induced DNA Damage

It has been suggested that Rad26-mediated repair of 4-nitroquinoline-1-oxide induced DNA damage is dependent on active transcription elongation by RNA Pol II, especially in the coding region of inducible genes [30]. To check this possibility in our yeast strain, we studied the effect of two transcription elongation inhibitors MPA and 6-AU on UV sensitivity of *RAD26-*deleted cells. Our results show that, when UV irradiated in presence of MPA or 6-AU, *RAD26-*deleted cells exhibit increased sensitivity compared to wild type (Fig. 2C & 2D). However, in absence of UV, sensitivity to either MPA or 6-AU is comparable in wild type and *RAD26-*deleted cells (Fig. 2A & 2B). This additional effect of *RAD26* deletion on UV sensitivity of MPA or 6-AU treated cells, i.e., where transcription elongation is adversely affected, indicates that Rad26-mediated TCR is not solely dependent on active transcription elongation, but rather on the presence of UV-induced DNA damage. It is therefore intriguing to conclude that when checked at the whole genome level, RNA polymerase II that gets stalled near a UV-induced DNA lesion probably requires Rad26 for subsequent TCR to take place. Since stalled RNA Pol II is known to obstruct DNA damage site, removal of the stalled RNAPII and/or remodeling of the DNA-RNAPII interface to allow access of NER factors to the lesion site must be an absolute requirement. Absence of Rad26 might impede such removal of RNAP II from near vicinity of DNA lesion, consequently affecting TCR and subsequent cell survival. Even in absence of active transcription elongation, any RNA Pol II that got loaded or had initiated transcription can occlude a UV-induced DNA lesion and subsequently call for Rad26-mediated TCR. This is in consonance with earlier suggestions that TCR may be initiated by loading of RNAPII, irrespective of transcription initiation or elongation [12], [13]. Indeed, it has been shown that in *GAL1* gene, loading of RNA pol II is sufficient to initiate Rad26-mediated TCR [13]. Taken together the results discussed above indicate that active transcription elongation is not a necessity for Rad26-mediated TCR of UV induced DNA lesions.

### Dissecting the Role of Rad26 during Transcription and TCR in RPB2 Locus

Considering the fact that cells lacking Rad26 have been reported to have transcription defects [14], we examined the expression of some of the major NER responsive genes in *RAD26*-deleted cells. Transcription analyses indicated that expression of the NER factors *RAD1*, *RAD2*, *RAD7*, *RAD16, RAD4, RAD23* and *RAD14* is not significantly affected in *RAD26*-deleted cells, in presence or absence of UV (Fig. 3A). We conclude that lack of Rad26 does not impair transcription of the NER genes tested. Consequently, the observed increased UV sensitivity of *RAD26*-deleted cells is not due to reduced expression of NER factors.

To further our understanding on the role of Rad26 in transcription, we next analyzed transcription of the constitutively active *RPB2* gene during NER. During repair following UV irradiation, we observed distinctly lowered transcription rate of *RPB2* gene in *RAD26*-deleted cells compared to wild type, especially after 60 min and 90 min of repair (Fig. 3B). This result indicates that lack of Rad26 affects TCR and subsequently the transcription of *RPB2* locus following repair. Thus, restoration of transcription following DNA damage repair requires presence of the TCR factor Rad26. As shown in our results, although absence of Rad26 delays resumption of *RPB2* gene transcription impaired due to UV-induced DNA damages, it does not influence transcription of *RPB2* under normal conditions (for e.g., in UV-untreated cells). This is in consonance with the recently published work of Bhaumik and his colleagues where they have shown that *RAD26* deletion does not affect transcription of a constitutively active gene like *RPS5* [31].

### Rad26 Helps to Remove Stalled RNAPII during DNA Damage Repair in Actively Transcribing Loci

To answer the next obvious question as to whether Rad26 has a role in removal of stalled RNAPII during TCR, ChIP based experiments were done. We found that during NER, both in wild type and H4 R45H cells, loss of RNA Pol II signal occurs in ORF1 and ORF2 located towards the 5′ end of the 3.4 kb *RPB2* locus (Figure 4 B and 4C). The loss and reappearance of the RNA Pol II signal occurs at earlier repair time points in the H4 R45H cells compared to wild type. Such early loss of RNA Pol II might be attributed to a more accessible chromatin landscape and higher transcriptional activity in H4R45H cells [26]. Our results of RNAPII disappearance is in consonance with the findings by Rockx and colleagues [32], who have shown in mammalian cells that transcription initiation is reduced by 1 hr post UV irradiation, concurrent with loss of the hypophosphorylated form of RNAPII (IIa) and reappearance of RNAPIIa after damage repair (6 hrs). The authors have suggested that regeneration of hypophosphorylated RNAPII after DNA damage repair plays an important role in restoration of transcription [32].

Most interestingly, we observed no significant loss of RNAPII during the repair incubations in either ORF1 or ORF2 of *RAD26*-deleted cells (Fig. 4B and 4C), indicating that Rad26 is required for removal of RNAPII in this region. Similar results were obtained when ChIP analyses were done in different ORF regions of two other constitutively transcribing loci namely, *PYK1* and *RPL2B* (Figure S2 & S3). Previous studies have suggested that in the *RPB2* locus, Rad26-mediated TCR plays a primary role compared to Rad26-independent TCR [3], [12], [33], [34]. Our results too indicate that during NER in the *RPB2* locus Rad26 works to remove stalled RNAPII. Recent studies have shown that yeast transcription elongation factors, Spt4 and Spt5 can cooperatively suppress TCR in absence of Rad26, but do not have any effect in Rad26-containing cells. Phosphorylated Spt5 is known to play a positive role in transcription by forming a stable complex with RNA Pol II and furthermore, overexpression of Spt5 seems to increase UV sensitivity of cells [35]. Keeping in mind the above findings it may be interesting to propose that when Spt5 forms a stable complex with RNAPII, Rad26 is essentially required to dissociate RNAPII for repair of a UV induced DNA lesion in the vicinity. Thus presence of Rad26 acts as an aid to disrupt the stable RNAPII-DNA complex whenever situations call for it.

**Figure 5:**
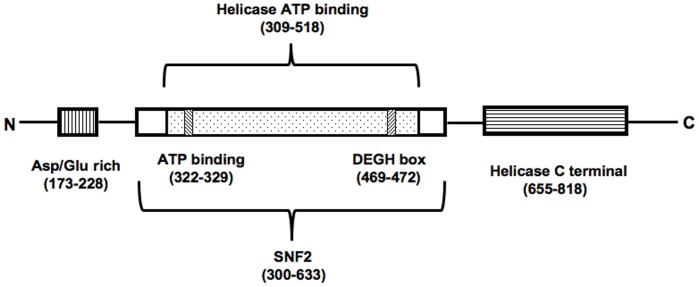
Domain organization of Rad26 protein. Bioinformatics based studies indicated the Rad26 protein to be composed of primarily three distinct domains. The N-terminal aspartate/glutamate-rich acidic domain; a SNF2 domain having the ATP-binding helicase sub-domain consisting of the ATP-binding pocket and a signature DEGH box; and the C-terminal helicase domain.

It has been further proposed that when arrested RNAPII leads to recruitment of Rad26, catalytic activity of Rad26, might pull the damaged DNA in the direction of the RNA polymerase or away from it, allowing the lesion to bulge out for repair [36], [37], [38]. In fact, as depicted in Figure 5, bioinformatics based studies of Rad26 protein revealed that the Snf2 domain in Rad26, includes a DEGH box. The DEGH box is a signature sequence known to be present in the TBP binding protein, BTAF1. TAF1, a member of the DNA-dependent SWI/SNF ATPase family, regulates transcription in association with TATA-binding protein (TBP), by removing TBP from the TATA box in an ATP-dependent manner (Pereira et al., 2003). The C-terminal ATPase domain of TAF1 which bears the signature DEGH box is essential for its DNA translocase activity by virtue of which it removes TBP from the TATA binding box or from AT-rich non-promoter sequences [39], [40]. In an analogous manner it is possible that, Rad26 which is a member of the DNA-dependent ATPase family, by virtue of the ATP- binding helicase domain with signature DEGH box (Fig. 5), might act as a translocase to change the RNAPII-DNA context and expose the damage site for subsequent NER.

Work by Woudstra et al., suggest that in cells lacking Rad26, Def1 mediated degradation of RNAPII occur within 1 hr of repair via ubiquitin-mediated protein degradation pathway [24]. Our ChIP results with the *RPB2* locus however, do not show disappearance of RNAPII in *RAD26*-deleted cells within the repair period tested. It is possible that the fate of RNAPII depends on the local chromatin structure and transcriptional activity of the gene tested. Whether RNAPII-DNA interface at the lesion site will be changed, or whether RNAPII will be degraded for the subsequent TCR to take place, possibly depends on the lesion type and the chromatin context in which it is found. For example, the *RPB2* locus being constitutively expressed, the locus must possess a more open and dynamic chromatin structure. In such a chromatin landscape, degradation of the RNAPII complex might not be favored. Rather, displacement of RNAPII or alteration of the DNA-RNAPII interface mediated by Rad26 will possibly be the primary pathway to expose the lesion site. Indeed, it was suggested that for TCR in eukaryotes it would be beneficial and energy conserving to allow for continuation of RNA synthesis after DNA repair, especially for transcripts that are considerably large [41]. Thus, it is possible that in cells lacking Rad26, neither displacement of RNAPII takes place nor the Def1 mediated degradation gets activated in a constitutively expressed locus like *RPB2*, at least during the early hours of repair. This might be the cause for the increased UV sensitivity of the *RAD26*-deleted cells observed in our experiments (Figure 1). Whether Rad26-independent TCR has a role to play under such situation still remains to be investigated.

Taken together, our results suggest that increased UV sensitivity observed in *RAD26*-deleted yeast cells is caused by inefficient removal of stalled RNAPII from the UV-induced DNA damage site leading to impaired repair of DNA lesion and subsequent loss of transcription restoration. This work thus provides additional insight into the role of Rad26 in transcription-coupled NER and resumption of transcription in constitutively expressed regions of yeast genome, following removal of DNA lesion.

## Supporting Information

Figure S1 ****RNA polymerase II occupancy in different ORFs of the *RPB2* locus in absence of UV irradiation.****
ChIP analysis of RNA polymerase II occupancy in ORF1, ORF2 and ORF3 of the *RPB2* locus as depicted in (Fig. 4A). Chromatin was immunoprecipitated with 8WG16 antibody specific to RNA polymerase II, followed by quantitative PCR amplification using primers specific to ORF1, ORF2 and ORF3 of the *RPB2* locus in WT, H4 R45H, WTΔRad26 and H4R45HΔRad26 cells. The values given for ORF1, ORF2 and ORF3 are calculated by normalizing the ChIP -PCR signal with the input PCR signal. For each set, data represent the mean ±1 SD for four independent experiments.
(TIF)

Figure S2 **RNA polymerase II status during NER in different regions of the** ***PYK1*** **locus. A.** ChIP analysis of RNA polymerase II status during NER was done in three ORF regions of the *PYK1* locus. **B.** Cells were irradiated with 100 J/m^2^ UV and incubated for different repair times as indicated. Chromatin was immunoprecipitated with 8WG16 antibody followed by quantitative PCR amplification using primers specific to ORF1, ORF2 and ORF3 of the *PYK1* locus in WT, WTΔRad26, H4 R45H and H4R45HΔRad26 cells. The values given for ORF1, ORF2 and ORF3 are calculated by normalizing the ChIP -PCR signal with the input PCR signal. The value for UV untreated cells was set as 1.0. For each strain, data represent the mean ±1 SD for three independent experiments.
(TIF)

Figure S3 **RNA polymerase II status during NER in different regions of the** ***RPL2B*** **locus. A.** ChIP analysis of RNA polymerase II status during NER was done in three ORF regions of the *RPL2B* locus. **B.** Cells were irradiated with 100 J/m^2^ UV and incubated for different repair times as indicated. Chromatin was immunoprecipitated with 8WG16 antibody followed by quantitative PCR amplification using primers specific to ORF1, ORF2 and ORF3 of the *RPL2B* locus in WT, WTΔRad26, H4 R45H and H4R45HΔRad26 cells. The values given for ORF1, ORF2 and ORF3 are calculated by normalizing the ChIP -PCR signal with the input PCR signal. The value for UV untreated cells was set as 1.0. For each strain, data represent the mean ±1 SD for three independent experiments.
(TIF)
