# Correction: Rad26, the Transcription-Coupled Repair Factor in Yeast, Is Required for Removal of Stalled RNA Polymerase-II following UV Irradiation

**DOI:** 10.1371/annotation/0625b456-da2e-44bd-b620-39796216cc2a

**Published:** 2013-09-13

**Authors:** Sounak Ghosh-Roy, Dhiman Das, Debarati Chowdhury, Michael J. Smerdon, Ronita Nag Chaudhuri

The was an error in presentation of the name of the fourth author.

The correct author name is: Michael J. Smerdon

The correct citation is: Ghosh-Roy S, Das D, Chowdhury D, Smerdon MJ, Chaudhuri RN (2013) Rad26, the Transcription-Coupled Repair Factor in Yeast, Is Required for Removal of Stalled RNA Polymerase-II following UV Irradiation. PLoS ONE 8(8): e72090. doi:10.1371/journal.pone.0072090 

