# Supplementary figures and images for: Rad26, the Transcription-Coupled Repair Factor in Yeast, Is Required for Removal of Stalled RNA Polymerase-II following UV Irradiation
Source: PLoS One. 2013 Aug 21;8(8):e72090. doi: 10.1371/journal.pone.0072090 (PMC3749123; doi:10.1371/journal.pone.0072090)

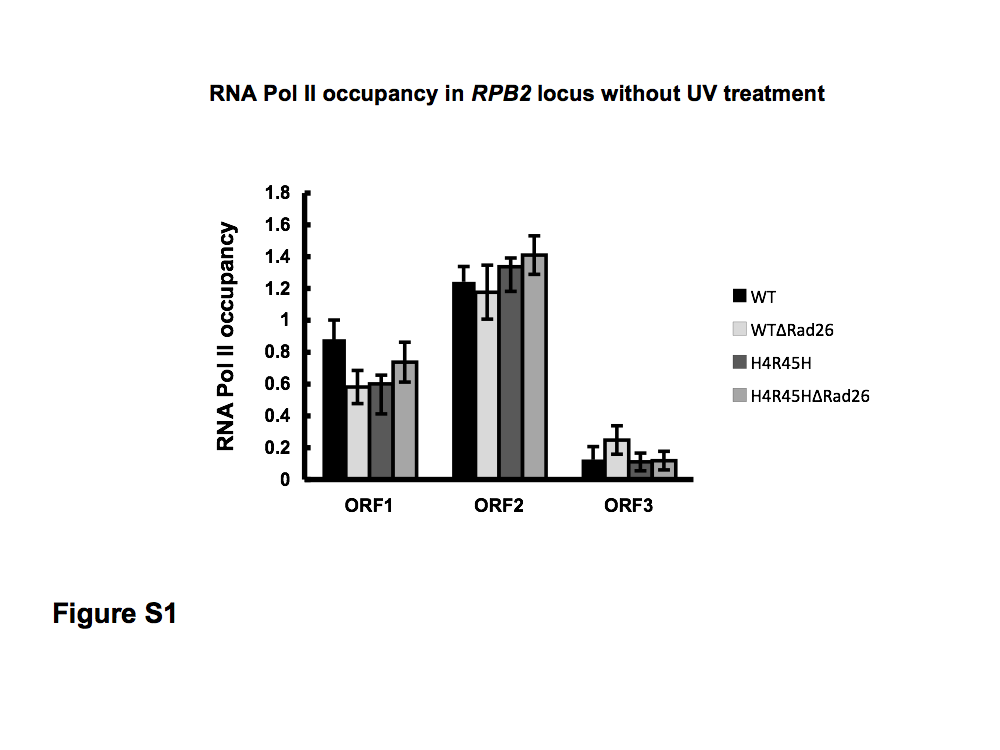

Supplement: Figure S1 — RNA polymerase II occupancy in different ORFs of the RPB2 locus in absence of UV irradiation. ChIP analysis of RNA polymerase II occupancy in ORF1, ORF2 and ORF3 of the RPB2 locus as depicted in (Fig. 4A). Chromatin was immunoprecipitated with 8WG16 antibody specific to RNA polymerase II, followed by quantitative PCR amplification using primers specific to ORF1, ORF2 and ORF3 of the RPB2 locus in WT, H4 R45H, WTΔRad26 and H4R45HΔRad26 cells. The values given for ORF1, ORF2 and ORF3 are calculated by normalizing the ChIP -PCR signal with the input PCR signal. For each set, data represent the mean ±1 SD for four independent experiments. (TIF) [file pone.0072090.s001.tif]

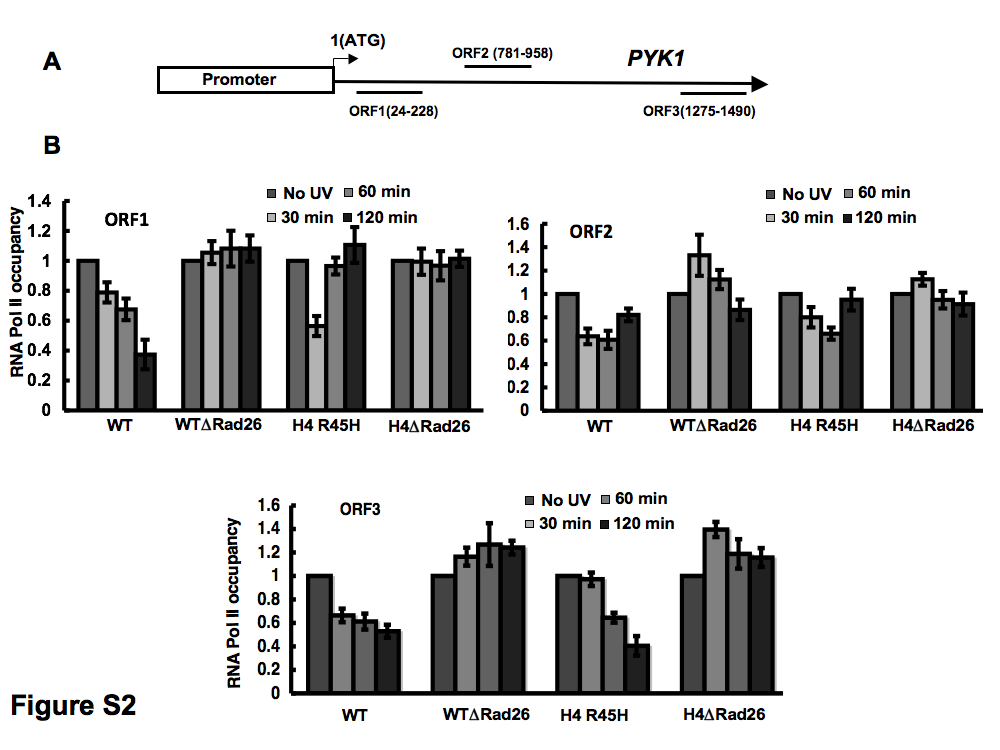

Supplement: Figure S2 — RNA polymerase II status during NER in different regions of the PYK1 locus. A. ChIP analysis of RNA polymerase II status during NER was done in three ORF regions of the PYK1 locus. B. Cells were irradiated with 100 J/m2 UV and incubated for different repair times as indicated. Chromatin was immunoprecipitated with 8WG16 antibody followed by quantitative PCR amplification using primers specific to ORF1, ORF2 and ORF3 of the PYK1 locus in WT, WTΔRad26, H4 R45H and H4R45HΔRad26 cells. The values given for ORF1, ORF2 and ORF3 are calculated by normalizing the ChIP -PCR signal with the input PCR signal. The value for UV untreated cells was set as 1.0. For each strain, data represent the mean ±1 SD for three independent experiments. (TIF) [file pone.0072090.s002.tif]

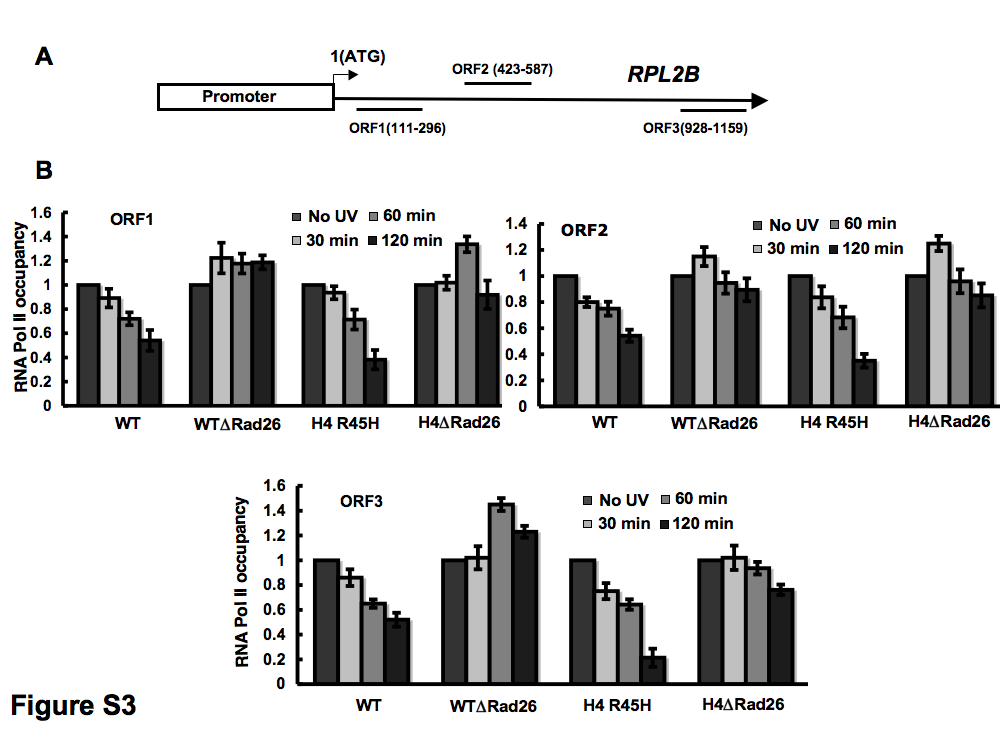

Supplement: Figure S3 — RNA polymerase II status during NER in different regions of the RPL2B locus. A. ChIP analysis of RNA polymerase II status during NER was done in three ORF regions of the RPL2B locus. B. Cells were irradiated with 100 J/m2 UV and incubated for different repair times as indicated. Chromatin was immunoprecipitated with 8WG16 antibody followed by quantitative PCR amplification using primers specific to ORF1, ORF2 and ORF3 of the RPL2B locus in WT, WTΔRad26, H4 R45H and H4R45HΔRad26 cells. The values given for ORF1, ORF2 and ORF3 are calculated by normalizing the ChIP -PCR signal with the input PCR signal. The value for UV untreated cells was set as 1.0. For each strain, data represent the mean ±1 SD for three independent experiments. (TIF) [file pone.0072090.s003.tif]
